# (2,2′-Bipyridine-κ^2^ *N*,*N*′)bis­(4-methyl­benzoato-κ^2^ *O*,*O*′)zinc(II)

**DOI:** 10.1107/S1600536808009483

**Published:** 2008-04-10

**Authors:** Yan-Qiu Shao

**Affiliations:** a Department of Chemistry, Mudanjiang Normal College, Mudanjiang 157012, People’s Republic of China

## Abstract

In the title compound, [Zn(C_8_H_7_O_2_)_2_(C_10_H_8_N_2_)], the Zn^II^ atom is coordinated by four O atoms from two chelating 4-methyl­benzoate ligands and two N atoms from a 2,2′-bipyridine ligand, displaying a disordered octahedral geometry. C—H⋯O hydrogen bonds connect the complex mol­ecules into a three-dimensional supra­molecular structure.

## Related literature

For related literature, see: Choi & Jeon (2003 ▶); Guilera & Steed (1999 ▶); Tao *et al.* (2000 ▶).
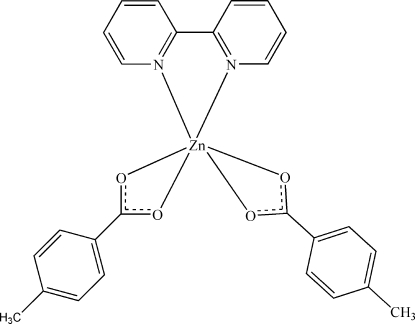


## Experimental

### 

#### Crystal data

[Zn(C_8_H_7_O_2_)_2_(C_10_H_8_N_2_)]
*M* *_r_* = 491.83
Triclinic, 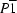

*a* = 7.6172 (3) Å
*b* = 9.8211 (4) Å
*c* = 15.7595 (6) Å
α = 79.130 (2)°
β = 83.977 (2)°
γ = 79.958 (2)°
*V* = 1136.90 (8) Å^3^
*Z* = 2
Mo *K*α radiation
μ = 1.12 mm^−1^
*T* = 296 (2) K
0.26 × 0.23 × 0.21 mm

#### Data collection

Bruker SMART APEXII CCD area-detector diffractometer
Absorption correction: multi-scan (*SADABS*; Bruker, 2001 ▶) *T*_min_ = 0.760, *T*_max_ = 0.799
10295 measured reflections
4047 independent reflections
3281 reflections with *I* > 2σ(*I*)
*R*_int_ = 0.030

#### Refinement

*R*[*F* ^2^ > 2σ(*F* ^2^)] = 0.035
*wR*(*F* ^2^) = 0.087
*S* = 1.02
4047 reflections
300 parameters
H-atom parameters constrained
Δρ_max_ = 0.22 e Å^−3^
Δρ_min_ = −0.29 e Å^−3^

### 

Data collection: *APEX2* (Bruker, 2007 ▶); cell refinement: *SAINT* (Bruker, 2007 ▶); data reduction: *SAINT*; program(s) used to solve structure: *SHELXS97* (Sheldrick, 2008 ▶); program(s) used to refine structure: *SHELXL97* (Sheldrick, 2008 ▶); molecular graphics: *SHELXTL* (Sheldrick, 2008 ▶); software used to prepare material for publication: *SHELXTL*.

## Supplementary Material

Crystal structure: contains datablocks I, global. DOI: 10.1107/S1600536808009483/hy2125sup1.cif

Structure factors: contains datablocks I. DOI: 10.1107/S1600536808009483/hy2125Isup2.hkl

Additional supplementary materials:  crystallographic information; 3D view; checkCIF report

## Figures and Tables

**Table:** 

N1—Zn1	2.090 (2)
N2—Zn1	2.103 (2)
O1—Zn1	2.509 (2)
O2—Zn1	1.9852 (18)
O3—Zn1	2.0626 (17)
O4—Zn1	2.2058 (19)

**Table:** 

O1—Zn1—O2	57.03 (7)
O1—Zn1—O3	95.86 (7)
O1—Zn1—O4	103.13 (7)
O1—Zn1—N1	92.92 (7)
O1—Zn1—N2	155.41 (8)
O2—Zn1—O3	145.10 (8)
O2—Zn1—N1	105.58 (8)
O3—Zn1—N1	96.58 (8)
O2—Zn1—N2	103.12 (8)
O3—Zn1—N2	107.73 (8)
N1—Zn1—N2	77.88 (9)
O2—Zn1—O4	100.93 (7)
O3—Zn1—O4	61.19 (7)
N1—Zn1—O4	153.43 (8)
N2—Zn1—O4	94.46 (8)

**Table 2:** Hydrogen-bond geometry (Å, °)

*D*—H⋯*A*	*D*—H	H⋯*A*	*D*⋯*A*	*D*—H⋯*A*
C3—H3⋯O3^i^	0.93	2.53	3.293 (3)	139
C4—H4⋯O1^i^	0.93	2.48	3.385 (3)	165
C14—H14⋯O4^ii^	0.93	2.51	3.417 (3)	164
C15—H15⋯O2^ii^	0.93	2.57	3.395 (3)	148
C20—H20⋯O3^iii^	0.93	2.43	3.189 (4)	139
C23—H23⋯O2^iv^	0.93	2.56	3.230 (4)	129

Symmetry codes: (i) 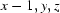; (ii) 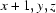; (iii) 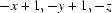; (iv) 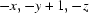.

**Fig. 1.**
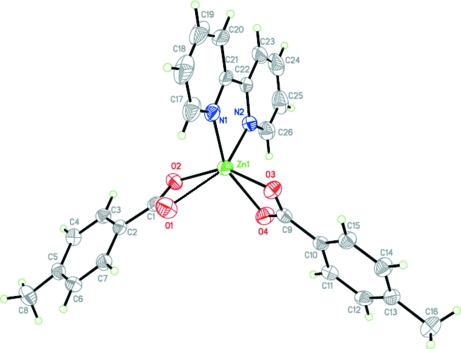
The molecular structure of the title compound. Displacement ellipsoids are drawn at the 30% probability level.

## supplementary crystallographic information

### Comment

Molecular self-assembly of supramolecular architectures has received much attention during recent decades (Tao *et al.*, 2000; Choi & Jeon, 2003). The structures and properties of such systems depend on the coordination and geometric preferences of both the central metal ions and the bridging building blocks, as well as on the influence of weaker non-covalent interactions, such as hydrogen bonds and π–π stacking interactions. As a building block, 4-methylbenzoate is an excellent candidate for the construction of supramolecular complexes. Recently, we obtained the title mononuclear complex by the reaction of cadmium chloride with 4-methylbenzoic acid and 2,2'-bipyridine in an aqueous solution and its crystal structure is reported here.

As depicted in Fig. 1, the Zn^II^ atom is coordinated by four O atoms from two 4-methylbenzoate ligands and two N atoms from one 2,2'-bipyridine ligand. The 4-methylbenzoates act as bidentate chelating ligands. The Zn1—O1 distance of 2.509 (2) Å (Table 1) suggests a non-negligible interaction, or a chelating coordination mode (Guilera & Steed, 1999). The complex molecules are connected by C—H···O hydrogen bonds (Table 2), resulting in a three-dimensional supramolecular network.

### Experimental

The title compound was prepared by the addition of a stoichiometric amount of cadmium chloride (0.228 g, 1 mmol) and 2,2'-bipyridine (0.156 g, 1 mmol) to a hot aqueous solution (25 ml) of 4-methylbenzoic acid (2.72 g, 20 mmol). The pH value was then adjusted to 7.0 to 8.0 with NaOH (1 mmol). The resulting solution was filtered, and colorless single crystals were obtained at room temperature over several days.

### Refinement

H atoms were positioned geometrically and refined as riding atoms, with C—H = 0.93 Å and *U*_iso_(H) = 1.2*U*_eq_(C) for aromatic groups and C—H = 0.96 Å and *U*_iso_(H) = 1.5*U*_eq_(C) for methyl groups.

### Crystal data

**Table:**

[Zn(C_8_H_7_O_2_)_2_(C_10_H_8_N_2_)]	*Z* = 2
*M**_r_* = 491.83	*F*_000_ = 508
Triclinic, *P*1	*D*_x_ = 1.437 Mg m^−^^3^
Hall symbol: -P 1	Mo *K*α radiation λ = 0.71073 Å
*a* = 7.6172 (3) Å	Cell parameters from 3500 reflections
*b* = 9.8211 (4) Å	θ = 1.3–28.0º
*c* = 15.7595 (6) Å	µ = 1.12 mm^−^^1^
α = 79.130 (2)º	*T* = 296 (2) K
β = 83.977 (2)º	Block, colorless
γ = 79.958 (2)º	0.26 × 0.23 × 0.21 mm
*V* = 1136.90 (8) Å^3^	

### Data collection

**Table:**

Bruker SMART APEXII CCD area-detector diffractometer	4047 independent reflections
Radiation source: fine-focus sealed tube	3281 reflections with *I* > 2σ(*I*)
Monochromator: graphite	*R*_int_ = 0.030
*T* = 296(2) K	θ_max_ = 25.2º
φ and ω scans	θ_min_ = 2.1º
Absorption correction: multi-scan(SADABS; Bruker, 2001)	*h* = −9→9
*T*_min_ = 0.760, *T*_max_ = 0.800	*k* = −11→11
10295 measured reflections	*l* = −18→18

### Refinement

**Table:**

Refinement on *F*^2^	Secondary atom site location: difference Fourier map
Least-squares matrix: full	Hydrogen site location: inferred from neighbouring sites
*R*[*F*^2^ > 2σ(*F*^2^)] = 0.035	H-atom parameters constrained
*wR*(*F*^2^) = 0.087	*w* = 1/[σ^2^(*F*_o_^2^) + (0.0378*P*)^2^ + 0.345*P*] where *P* = (*F*_o_^2^ + 2*F*_c_^2^)/3
*S* = 1.02	(Δ/σ)_max_ = 0.001
4047 reflections	Δρ_max_ = 0.22 e Å^−^^3^
300 parameters	Δρ_min_ = −0.29 e Å^−^^3^
Primary atom site location: structure-invariant direct methods	Extinction correction: none

### Fractional atomic coordinates and isotropic or equivalent isotropic displacement parameters (Å^2^)

**Table:**

	*x*	*y*	*z*	*U*_iso_*/*U*_eq_	
C1	−0.0510 (3)	0.7966 (3)	0.21798 (15)	0.0470 (6)	
C2	−0.2171 (3)	0.8766 (2)	0.25555 (15)	0.0411 (6)	
C3	−0.3837 (3)	0.8465 (3)	0.24538 (16)	0.0453 (6)	
H3	−0.3925	0.7784	0.2130	0.054*	
C4	−0.5367 (3)	0.9160 (3)	0.28257 (17)	0.0487 (6)	
H4	−0.6476	0.8966	0.2731	0.058*	
C5	−0.5282 (4)	1.0141 (3)	0.33374 (17)	0.0497 (6)	
C6	−0.3604 (4)	1.0445 (3)	0.34364 (17)	0.0531 (7)	
H6	−0.3512	1.1105	0.3775	0.064*	
C7	−0.2073 (4)	0.9783 (3)	0.30400 (16)	0.0486 (6)	
H7	−0.0970	1.0023	0.3099	0.058*	
C8	−0.6941 (4)	1.0840 (3)	0.3795 (2)	0.0725 (9)	
H8A	−0.7971	1.0515	0.3650	0.109*	
H8B	−0.7059	1.1839	0.3614	0.109*	
H8C	−0.6848	1.0610	0.4409	0.109*	
C9	0.3993 (3)	0.4970 (3)	0.29191 (16)	0.0446 (6)	
C10	0.5328 (3)	0.4398 (2)	0.35701 (15)	0.0404 (6)	
C11	0.4819 (4)	0.3697 (3)	0.43803 (16)	0.0455 (6)	
H11	0.3631	0.3584	0.4522	0.055*	
C12	0.6075 (4)	0.3164 (3)	0.49798 (16)	0.0517 (7)	
H12	0.5715	0.2695	0.5522	0.062*	
C13	0.7847 (4)	0.3311 (3)	0.47934 (17)	0.0513 (7)	
C14	0.8342 (4)	0.4020 (3)	0.39820 (18)	0.0566 (7)	
H14	0.9531	0.4132	0.3842	0.068*	
C15	0.7102 (3)	0.4562 (3)	0.33780 (16)	0.0505 (7)	
H15	0.7461	0.5042	0.2838	0.061*	
C16	0.9214 (5)	0.2747 (4)	0.5456 (2)	0.0805 (10)	
H16A	0.8629	0.2348	0.5992	0.121*	
H16B	0.9785	0.3498	0.5550	0.121*	
H16C	1.0096	0.2038	0.5247	0.121*	
C17	0.3103 (4)	0.8005 (4)	0.0171 (2)	0.0748 (9)	
H17	0.3061	0.8620	0.0558	0.090*	
C18	0.3617 (5)	0.8439 (5)	−0.0679 (3)	0.1067 (16)	
H18	0.3889	0.9339	−0.0870	0.128*	
C19	0.3723 (5)	0.7545 (6)	−0.1234 (3)	0.118 (2)	
H19	0.4101	0.7814	−0.1813	0.142*	
C20	0.3270 (4)	0.6220 (5)	−0.0949 (2)	0.0895 (13)	
H20	0.3328	0.5596	−0.1331	0.107*	
C21	0.2722 (3)	0.5840 (3)	−0.00735 (16)	0.0585 (8)	
C22	0.2212 (3)	0.4456 (3)	0.03117 (18)	0.0554 (7)	
C23	0.2133 (5)	0.3418 (5)	−0.0157 (2)	0.0829 (11)	
H23	0.2405	0.3565	−0.0753	0.099*	
C24	0.1654 (5)	0.2173 (5)	0.0259 (3)	0.0984 (15)	
H24	0.1584	0.1474	−0.0054	0.118*	
C25	0.1274 (4)	0.1953 (4)	0.1141 (3)	0.0843 (11)	
H25	0.0972	0.1102	0.1435	0.101*	
C26	0.1355 (4)	0.3035 (3)	0.1576 (2)	0.0640 (8)	
H26	0.1088	0.2901	0.2173	0.077*	
N1	0.2659 (3)	0.6739 (2)	0.04704 (14)	0.0531 (6)	
N2	0.1801 (3)	0.4265 (2)	0.11749 (13)	0.0491 (5)	
O1	0.0937 (3)	0.8418 (2)	0.21029 (14)	0.0713 (6)	
O2	−0.0627 (2)	0.68095 (19)	0.19582 (11)	0.0527 (5)	
O3	0.4440 (2)	0.57256 (18)	0.22078 (11)	0.0525 (5)	
O4	0.2425 (2)	0.4705 (2)	0.30517 (12)	0.0621 (5)	
Zn1	0.19260 (4)	0.59907 (3)	0.176739 (18)	0.04603 (12)	

### Atomic displacement parameters (Å^2^)

**Table:**

	*U*^11^	*U*^22^	*U*^33^	*U*^12^	*U*^13^	*U*^23^
C1	0.0471 (16)	0.0555 (18)	0.0358 (13)	−0.0043 (13)	−0.0065 (11)	−0.0032 (12)
C2	0.0455 (15)	0.0373 (14)	0.0384 (13)	−0.0039 (11)	−0.0066 (11)	−0.0020 (11)
C3	0.0517 (16)	0.0425 (15)	0.0434 (14)	−0.0104 (12)	−0.0069 (12)	−0.0071 (12)
C4	0.0411 (15)	0.0476 (16)	0.0574 (16)	−0.0115 (12)	−0.0029 (12)	−0.0055 (13)
C5	0.0540 (17)	0.0377 (15)	0.0523 (15)	−0.0027 (12)	0.0000 (13)	−0.0018 (12)
C6	0.0627 (18)	0.0482 (16)	0.0517 (16)	−0.0059 (14)	−0.0093 (13)	−0.0170 (13)
C7	0.0457 (15)	0.0478 (16)	0.0545 (16)	−0.0062 (12)	−0.0113 (12)	−0.0117 (13)
C8	0.063 (2)	0.064 (2)	0.088 (2)	−0.0048 (16)	0.0114 (17)	−0.0212 (18)
C9	0.0499 (16)	0.0399 (15)	0.0460 (15)	−0.0036 (12)	−0.0021 (12)	−0.0166 (12)
C10	0.0483 (15)	0.0362 (14)	0.0381 (13)	−0.0069 (11)	−0.0014 (11)	−0.0104 (11)
C11	0.0501 (15)	0.0447 (15)	0.0435 (14)	−0.0115 (12)	0.0036 (12)	−0.0126 (12)
C12	0.0713 (19)	0.0448 (16)	0.0380 (14)	−0.0110 (14)	0.0028 (13)	−0.0072 (12)
C13	0.0602 (18)	0.0475 (16)	0.0468 (15)	−0.0029 (13)	−0.0108 (13)	−0.0108 (13)
C14	0.0454 (16)	0.0673 (19)	0.0563 (17)	−0.0121 (14)	−0.0045 (13)	−0.0052 (15)
C15	0.0521 (17)	0.0554 (17)	0.0419 (14)	−0.0128 (13)	−0.0012 (12)	−0.0005 (12)
C16	0.083 (2)	0.091 (3)	0.065 (2)	−0.002 (2)	−0.0273 (18)	−0.0045 (18)
C17	0.0556 (19)	0.076 (2)	0.075 (2)	−0.0037 (16)	0.0001 (16)	0.0213 (18)
C18	0.065 (2)	0.118 (4)	0.095 (3)	0.006 (2)	0.015 (2)	0.054 (3)
C19	0.071 (3)	0.170 (5)	0.060 (2)	0.035 (3)	0.019 (2)	0.051 (3)
C20	0.065 (2)	0.137 (4)	0.0404 (17)	0.038 (2)	0.0007 (15)	−0.005 (2)
C21	0.0404 (15)	0.085 (2)	0.0358 (14)	0.0209 (15)	−0.0045 (11)	−0.0023 (15)
C22	0.0424 (15)	0.071 (2)	0.0486 (16)	0.0188 (14)	−0.0149 (12)	−0.0199 (15)
C23	0.076 (2)	0.099 (3)	0.077 (2)	0.033 (2)	−0.0329 (19)	−0.052 (2)
C24	0.082 (3)	0.091 (3)	0.139 (4)	0.026 (2)	−0.050 (3)	−0.076 (3)
C25	0.066 (2)	0.060 (2)	0.134 (4)	−0.0004 (17)	−0.028 (2)	−0.033 (2)
C26	0.0607 (19)	0.0565 (19)	0.077 (2)	−0.0072 (15)	−0.0087 (15)	−0.0156 (17)
N1	0.0446 (13)	0.0597 (15)	0.0449 (13)	0.0013 (11)	0.0001 (10)	0.0051 (12)
N2	0.0439 (13)	0.0557 (14)	0.0469 (13)	−0.0007 (10)	−0.0046 (10)	−0.0127 (11)
O1	0.0465 (12)	0.0895 (16)	0.0860 (15)	−0.0154 (11)	−0.0004 (10)	−0.0336 (13)
O2	0.0564 (12)	0.0468 (11)	0.0540 (11)	−0.0047 (9)	0.0036 (9)	−0.0144 (9)
O3	0.0564 (12)	0.0522 (11)	0.0483 (10)	−0.0132 (9)	−0.0096 (9)	−0.0004 (9)
O4	0.0450 (11)	0.0880 (15)	0.0530 (11)	−0.0139 (10)	−0.0029 (9)	−0.0089 (10)
Zn1	0.0458 (2)	0.0512 (2)	0.04008 (18)	−0.00641 (14)	−0.00186 (12)	−0.00715 (13)

### Geometric parameters (Å, °)

**Table:**

C1—O1	1.244 (3)	C16—H16A	0.9600
C1—O2	1.270 (3)	C16—H16B	0.9600
C1—C2	1.496 (4)	C16—H16C	0.9600
C2—C7	1.383 (3)	C17—N1	1.332 (4)
C2—C3	1.384 (3)	C17—C18	1.366 (5)
C3—C4	1.378 (4)	C17—H17	0.9300
C3—H3	0.9300	C18—C19	1.339 (6)
C4—C5	1.381 (4)	C18—H18	0.9300
C4—H4	0.9300	C19—C20	1.384 (6)
C5—C6	1.393 (4)	C19—H19	0.9300
C5—C8	1.510 (4)	C20—C21	1.398 (4)
C6—C7	1.382 (4)	C20—H20	0.9300
C6—H6	0.9300	C21—N1	1.334 (4)
C7—H7	0.9300	C21—C22	1.480 (4)
C8—H8A	0.9600	C22—N2	1.349 (3)
C8—H8B	0.9600	C22—C23	1.380 (4)
C8—H8C	0.9600	C23—C24	1.364 (6)
C9—O4	1.254 (3)	C23—H23	0.9300
C9—O3	1.272 (3)	C24—C25	1.372 (6)
C9—C10	1.485 (3)	C24—H24	0.9300
C10—C11	1.383 (3)	C25—C26	1.383 (4)
C10—C15	1.384 (3)	C25—H25	0.9300
C11—C12	1.382 (4)	C26—N2	1.336 (4)
C11—H11	0.9300	C26—H26	0.9300
C12—C13	1.377 (4)	N1—Zn1	2.090 (2)
C12—H12	0.9300	N2—Zn1	2.103 (2)
C13—C14	1.384 (4)	O1—Zn1	2.509 (2)
C13—C16	1.514 (4)	O2—Zn1	1.9852 (18)
C14—C15	1.380 (4)	O3—Zn1	2.0626 (17)
C14—H14	0.9300	O4—Zn1	2.2058 (19)
C15—H15	0.9300		
			
O1—C1—O2	121.5 (3)	N1—C17—C18	122.8 (4)
O1—C1—C2	120.6 (2)	N1—C17—H17	118.6
O2—C1—C2	117.8 (2)	C18—C17—H17	118.6
C7—C2—C3	118.5 (2)	C19—C18—C17	118.8 (4)
C7—C2—C1	120.8 (2)	C19—C18—H18	120.6
C3—C2—C1	120.7 (2)	C17—C18—H18	120.6
C4—C3—C2	121.0 (2)	C18—C19—C20	120.2 (4)
C4—C3—H3	119.5	C18—C19—H19	119.9
C2—C3—H3	119.5	C20—C19—H19	119.9
C3—C4—C5	121.1 (2)	C19—C20—C21	118.6 (4)
C3—C4—H4	119.5	C19—C20—H20	120.7
C5—C4—H4	119.5	C21—C20—H20	120.7
C4—C5—C6	117.8 (2)	N1—C21—C20	120.1 (3)
C4—C5—C8	121.5 (3)	N1—C21—C22	116.0 (2)
C6—C5—C8	120.7 (3)	C20—C21—C22	123.8 (3)
C7—C6—C5	121.1 (2)	N2—C22—C23	121.0 (3)
C7—C6—H6	119.4	N2—C22—C21	115.0 (2)
C5—C6—H6	119.4	C23—C22—C21	124.0 (3)
C6—C7—C2	120.4 (2)	C24—C23—C22	119.6 (4)
C6—C7—H7	119.8	C24—C23—H23	120.2
C2—C7—H7	119.8	C22—C23—H23	120.2
C5—C8—H8A	109.5	C23—C24—C25	120.0 (3)
C5—C8—H8B	109.5	C23—C24—H24	120.0
H8A—C8—H8B	109.5	C25—C24—H24	120.0
C5—C8—H8C	109.5	C24—C25—C26	117.9 (4)
H8A—C8—H8C	109.5	C24—C25—H25	121.0
H8B—C8—H8C	109.5	C26—C25—H25	121.0
O4—C9—O3	118.9 (2)	N2—C26—C25	122.6 (3)
O4—C9—C10	121.1 (2)	N2—C26—H26	118.7
O3—C9—C10	119.9 (2)	C25—C26—H26	118.7
C11—C10—C15	118.8 (2)	C17—N1—C21	119.5 (3)
C11—C10—C9	120.6 (2)	C17—N1—Zn1	124.7 (2)
C15—C10—C9	120.6 (2)	C21—N1—Zn1	115.76 (18)
C12—C11—C10	120.1 (2)	C26—N2—C22	118.8 (3)
C12—C11—H11	120.0	C26—N2—Zn1	125.87 (19)
C10—C11—H11	120.0	C22—N2—Zn1	115.32 (19)
C13—C12—C11	121.6 (2)	C1—O2—Zn1	101.97 (16)
C13—C12—H12	119.2	C9—O3—Zn1	92.93 (15)
C11—C12—H12	119.2	C9—O4—Zn1	86.93 (15)
C12—C13—C14	117.9 (2)	O1—Zn1—O2	57.03 (7)
C12—C13—C16	121.5 (3)	O1—Zn1—O3	95.86 (7)
C14—C13—C16	120.6 (3)	O1—Zn1—O4	103.13 (7)
C15—C14—C13	121.1 (3)	O1—Zn1—N1	92.92 (7)
C15—C14—H14	119.4	O1—Zn1—N2	155.41 (8)
C13—C14—H14	119.4	O2—Zn1—O3	145.10 (8)
C14—C15—C10	120.4 (2)	O2—Zn1—N1	105.58 (8)
C14—C15—H15	119.8	O3—Zn1—N1	96.58 (8)
C10—C15—H15	119.8	O2—Zn1—N2	103.12 (8)
C13—C16—H16A	109.5	O3—Zn1—N2	107.73 (8)
C13—C16—H16B	109.5	N1—Zn1—N2	77.88 (9)
H16A—C16—H16B	109.5	O2—Zn1—O4	100.93 (7)
C13—C16—H16C	109.5	O3—Zn1—O4	61.19 (7)
H16A—C16—H16C	109.5	N1—Zn1—O4	153.43 (8)
H16B—C16—H16C	109.5	N2—Zn1—O4	94.46 (8)

### Hydrogen-bond geometry (Å, °)

**Table:**

*D*—H···*A*	*D*—H	H···*A*	*D*···*A*	*D*—H···*A*
C3—H3···O3^i^	0.93	2.53	3.293 (3)	139
C4—H4···O1^i^	0.93	2.48	3.385 (3)	165
C14—H14···O4^ii^	0.93	2.51	3.417 (3)	164
C15—H15···O2^ii^	0.93	2.57	3.395 (3)	148
C20—H20···O3^iii^	0.93	2.43	3.189 (4)	139
C23—H23···O2^iv^	0.93	2.56	3.230 (4)	129

Symmetry codes: (i) *x*−1, *y*, *z*; (ii) *x*+1, *y*, *z*; (iii) −*x*+1, −*y*+1, −*z*; (iv) −*x*, −*y*+1, −*z*.
